# *Mycobacterium lentiflavum* in Drinking Water Supplies, Australia

**DOI:** 10.3201/eid1703.090948

**Published:** 2011-03

**Authors:** Henry M. Marshall, Robyn Carter, Matthew J. Torbey, Sharri Minion, Carla Tolson, Hanna E. Sidjabat, Flavia Huygens, Megan Hargreaves, Rachel M. Thomson

**Affiliations:** Author affiliations: The Prince Charles Hospital, Brisbane, Queensland, Australia (H.M. Marshall);; The Royal Brisbane Hospital, Brisbane (R. Carter, M.J. Torbey, S. Minion, C. Tolson);; University of Queensland Centre for Clinical Research, Brisbane (H.E. Sidjabat);; Queensland University of Technology, Brisbane (F. Huygens, M. Hargreaves);; Queensland Tuberculosis Control Centre, Brisbane (R.M. Thomson)

**Keywords:** Bacteria, nontuberculous mycobacteria, genotyping, Mycobacterium lentiflavum, environmental microbiology, potable water supply, fresh water microbiology, Australia, tuberculosis and other mycobacteria, research

## Abstract

*Mycobacterium lentiflavum*, a slow-growing nontuberculous mycobacterium, is a rare cause of human disease. It has been isolated from environmental samples worldwide. To assess the clinical significance of *M. lentiflavum* isolates reported to the Queensland Tuberculosis Control Centre, Australia, during 2001–2008, we explored the genotypic similarity and geographic relationship between isolates from humans and potable water in the Brisbane metropolitan area. A total of 47 isolates from 36 patients were reported; 4 patients had clinically significant disease. *M. lentiflavum* was cultured from 13 of 206 drinking water sites. These sites overlapped geographically with home addresses of the patients who had clinically significant disease. Automated repetitive sequence–based PCR genotyping showed a dominant environmental clone closely related to clinical strains. This finding suggests potable water as a possible source of *M. lentiflavum* infection in humans.

## Abstract 2

Humans may acquire infection from potable water.

*Mycobacterium lentiflavum* organisms are nontuberculous mycobacteria (NTM) first identified in 1996 (*1*). *M. lentiflavum* is slow growing at 22°C–37°C and has yellow pigmentation, negative tests for Tween 80 hydrolysis, nicotinic acid, nitrate reductase and urease, distinct fatty and mycolic acid patterns, and unique 16S rRNA and 65-kDa heat-shock protein gene sequences. It shares phenotypic features with *M. avium* but is more closely related to *M. simiae* and *M. genavense*. Because of similarities to *M. avium* complex (MAC), differentiation can be difficult without molecular identification, hence, misclassification in the past is possible (*2*).

As with other NTM, *M. lentiflavum* has been isolated from soil and water samples around the world. However, links between environmental sources and human disease have not yet been demonstrated.

In Queensland, Australia (population 4.28 million), NTM disease is notifiable. A central reference laboratory performs speciation of all positive isolates. In 2008, ≈900 isolates of NTM were reported.

Strain variation within mycobacterial species is well known. Although epidemiologic studies provide useful information, molecular strain typing can be invaluable, especially if a single clone can be linked to an outbreak source. Pulsed-field gel electrophoresis (PFGE) has been considered the standard for mycobacterial strain typing but is time- and labor- intensive and requires expensive dedicated equipment. Also, DNA degradation can occur during electrophoresis, generating uninterpretable banding patterns (*3*). Repetitive sequence–based PCR (rep-PCR) has been used to differentiate mycobacterial strains associated with disease outbreaks in mesotherapy clinics (*M. abscessus* and *M. chelonae*) (*4*) and in patients after surgery (*M. fortuitum*) (*5*). An automated rep-PCR system (DiversiLab; bioMérieux, Melbourne, Victoria, Australia) showed high concordance with PFGE results (*6*) in identifying mycobacterial strain clusters and was faster than PFGE.

We had 2 goals for this study. First, we aimed to describe the clinical significance and outcomes of *M. lentiflavum* infection in Queensland. Second, we intended to explore the genotypic and geographic relationship between patient isolates and potable water isolates in the Brisbane area.

## Methods

We reviewed the records of all patients from whom *M. lentiflavum* had been isolated during July 2001–November 2008. Attending physicians were contacted to establish clinical significance according to American Thoracic Society (ATS)/Infectious Diseases Society of America (IDSA) criteria (*2*) (Table 1). During 2007–2008, potable water was collected from 206 sites in Brisbane’s drinking water system.

**Table 1:** American Thoracic Society/Infectious Diseases Society of America diagnostic criteria for NTM lung disease*

Clinical criteria
Pulmonary symptoms AND
Nodular or cavitary opacities on chest radiograph OR
Multifocal bronchiectasis with multiple small nodules on high-resolution computerized tomography AND
Appropriate exclusion of other diagnoses
Microbiologic
Positive culture results from at least 2 separate expectorated sputum samples OR
Positive culture results from at least 1 bronchial wash or lavage OR
Biopsy† showing granulomatous inflammation or acid-fast bacilli *and* positive culture OR
Biopsy† showing granulomatous inflammation or acid-fast bacilli *and* one or more culture-positive sputum or bronchial washings
Comments
• Risk-benefit of therapy should be considered for each patient • before institution of therapy
• Expert consultation should be obtained when NTM are • recovered that are either infrequently encountered or that • usually represent environmental contamination
• Patients suspected of having NTM lung disease but who do • not meet the diagnostic criteria should be followed until the • diagnosis is firmly established or excluded

*Adapted from (*2*). NTM, nontuberculous mycobacteria. †Transbronchial or other lung biopsy.

### Laboratory Identification

Human samples were digested and decontaminated by using 4% NaOH, neutralized with phosphoric acid, and centrifuged to concentrate the acid-fast bacilli (AFB). Smears were prepared from the sediment and stained by the Ziehl-Neelsen (ZN) method. We injected cells into 1 Lowenstein-Jensen slope (± pyruvate) and 7-mL mycobacterial growth indicator tube, then incubated them at 35°C until growth was detected. ZN staining of colonies confirmed AFB. Multiplex PCR (*7*) was performed to discriminate between *M. tuberculosis*, *M. avium*, *M. intracellulare*, *M. abscessus*, and other *Mycobacterium* spp. Other *Mycobacterium* spp. were further speciated by using Hain Life Sciences GenoType Mycobacterium AS (additional species) kit (2004–2007 only; Hain Lifescience, Nehren, Germany) and/or 16S rRNA sequencing in conjunction with phenotypic characteristics.

### Water Sampling

Water was collected from routine sampling sites across Brisbane and processed according to described methods (*8*). Each 1,000-mL sample was transported at 4°C and processed within 24 hours. Half of each sample was decontaminated by using 0.005% cetylpiridinium chloride, and each 500-mL aliquot was filtered separately by using 45-µm cellulose nitrate filters (Sartorius AG, Gottingen, Germany). The filters were rinsed and macerated in 3 mL sterile distilled water. Aliquots (0.1 mL) were transferred in triplicate to M7H11 plates, sealed in gas-permeable plastic bags, and incubated at 32°C. Aliquots (0.5 mL) were transferred to 2 mycobacterial growth indicator tubes, 1 of which contained polymyxin, azlocillin, nalidixic acid, trimethoprim, and amphotericin B. ZN staining of colonies confirmed AFB, and these colonies were subcultured on M7H11 plates. Multiplex PCR was performed (*7*) followed by 16S rRNA sequencing of mycobacterial isolates and compared by using Ribosomal Differentiation of Medical Microorganisms and GenBank databases (*9**,**10*).

### Automated Rep-PCR Strain Typing

The similarity of 16 clinical and 7 water isolates was determined by using a rep-PCR method (DiversiLab). DNA was extracted from clinical and water isolates by using the Ultraclean Microbial DNA Isolation Kit (Mobio Laboratories, Carlsbad, CA, USA). PCR mixture was prepared by using AmpliTaq polymerase and PCR buffer (Applied Biosystems, Foster City, CA, USA) and Mycobacterium DiversiLab primer mix according to the manufacturer’s instructions. Rep-PCR products were separated and detected by using microfluidic chips of the DiversiLab system. Fingerprints were analyzed with DiversiLab software version 3.4.38 by using the Pearson correlation coefficient and unweighted pair-group method with arithmetic means to compare isolates and determine clonal relationships.

## Results

### Clinical Isolates

**Figure 1:**
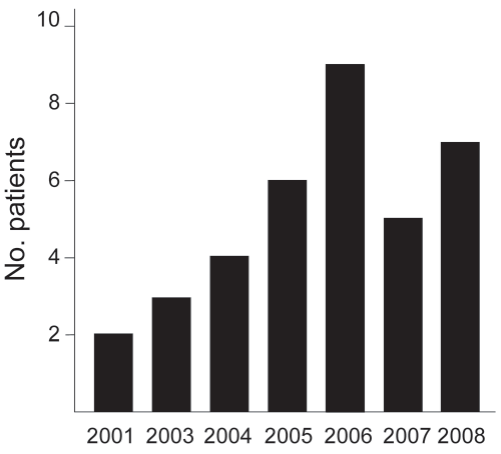
Number of patients from whom *Mycobacterium lentiflavum* was isolated, by year of isolation, Brisbane, Queensland, Australia, 2001–2008.

Forty-seven isolates of *M. lentiflavum* were reported from 36 patients (Figure 1; Table 2). Full clinical information was available for 32 (89%) patients. Four patients (8 isolates) had clinically significant disease. Seven patients were taking treatment or were under surveillance for MAC (1 or 2 isolates each); no treatment changed in response to the new isolate, and thus these isolates were not considered clinically significant. Twenty-one other patients (18 adults, 3 children) had clinically nonsignificant isolates. Four patients had probable nonsignificant isolates, but sufficient clinical information was lacking. No cases demonstrated positive AFB smears by ZN staining. Of the 32 patients with uncertain or nonsignificant disease, 26 had 1 positive specimen, 2 had 2 positive specimens from the same period, 3 had 2 positive specimens separated by 3 months, and 1 had 2 positive specimens separated by 11 months. Antimicrobial drug susceptibility tests were performed for 2 isolates (cases 1 and 2 below). Both were sensitive to clarithromycin 4.0 µg/mL and resistant to isoniazid 0.4 µg/mL, ethambutol 5.0 µg/mL, and streptomycin 1.0 µg/mL. The case 1 isolate was sensitive to ofloxacin 2.0 µg/mL; the case 2 isolate was resistant to rifampin 1.0 µg/mL.

**Table 2:** Characteristics of patients from whom *Mycobacterium lentiflavum* was isolated and source of isolate, Queensland, Australia, 2001–2008*

Characteristic	No. patients	Median age, y (range); sex, M/F	Source, no. isolates			
			Bronchial washing	Sputum	Wound swab/aspirate	Other
Adults						
Significant clinical illness	3	49 (42–85); 0/3	2	0	0	Blood, 1
Nonsignificant clinical illness	18	67 (22–88); 12/6	9	4	4	Blood, 1
Probable nonsignificant clinical illness	4	74 (59–81); 3/1	1	2	0	Ascites, 1
Nonsignificant clinical illness with MAC	7	66 (49–75); 4/3	0	7	0	0
Children						
Significant clinical illness	1	1.6; 0/1	0	0	1	0
Nonsignificant clinical illness	3	12 (1.6–17); 1/2	0	2	1	0

*MAC, *Mycobacterium avium* complex.

### Environmental Isolates

**Figure 2:**
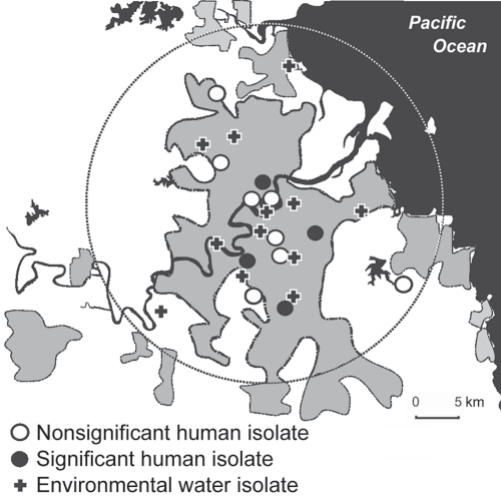
Urban catchment area and locations of persons and potable water from which *Mycobacterium lentiflavum* was isolated, Brisbane, Queensland, Australia, 2001–2008. Gray shading, approximate urban extent; circle, 20-km radius from central business district.

Mycobacteria were grown from 70% of water sites. The predominant isolates were *M*. *gordonae* and *M. kansasii*. *M. lentiflavum* was isolated from 13 (6.3%) sites, 2 of which were reservoirs, 1 a treatment plant, and the remainder points in the distribution system. Eleven sites shared the same groundwater source but were distributed among 10 different reservoir zones. For 12 patients living within 20 km of Brisbane central business district, the mean distance between their residential addresses and nearest positive water site was 3.49 km (range 0.9–9.8 km). The 4 persons with clinically significant illness lived a mean of 2.7 km from a positive water site (Figure 2).

### Case Descriptions for Significant Isolates

The 4 patients whose disease met the ATS/IDSA criteria are described below. All specimens were ZN stain negative.

#### Case 1: Disseminated Infection

A 43-year-old woman who smoked had a background of intravenous drug use and HIV. In 1998, granulomatous hepatomegaly developed, thought to be a reaction from injecting methadone mixed with orange juice, and resolved after she ceased this activity. A tunneled intravenous access device was placed in February 2006. In April 2007, she sought care for hepatosplenomegaly and mild pancytopenia. Liver and gastric lymph node biopsies showed granulomata. Two bone marrow biopsy samples taken 6 weeks apart showed initially scant, but then more marked, granulomata. All specimens were culture negative for AFB. A working diagnosis of sarcoidosis was made, and prednisone with highly active antiretroviral therapy (tenofovir, emtricitabine, and efavirenz) began. Azathioprine was introduced and prednisone ceased by April 2008. In June, she was admitted with massive hepatosplenomegaly, weight loss, and fever. CD4+ count was 0.14 × 10^9^/L (0.43–1.62 × 10^9^/L), and viral load was undetectable (<50 copies/mL HIV-1 RNA). Over the next month, all 4 blood cultures grew *M. lentiflavum*; after 15 days, mycobacteria were apparent and *M. lentiflavum* was confirmed 7 days later (day 22). Bone marrow biopsy showed granulomata and grew *M. lentiflavum*. Urine and fecal samples were negative for any mycobacteria. She did not produce any sputum. Chest radiograph showed extensive miliary nodules, and computed tomography (CT) showed peribronchial thickening and bronchiolitis but no lymphadenopathy. The patient was empirically given isoniazid, rifampicin, pyrazinamide, clarithromycin, and ethambutol. Oral prednisone (25 mg 1×/d) improved symptoms and liver biochemistry and decreased splenic size. She was discharged on prednisone (15 mg 1×/d), isoniazid (300 mg 1×/d), ethambutol (400 mg 2×/d), and clarithromycin (500 mg 2×/d). Her organomegaly improved over the next 6 months. The intravenous port was removed. Ten months later, she remained well and compliant with treatment.

#### Case 2: Chronic Pulmonary Nodules and Bronchiectasis

In December 2007, an 85-year-old woman sought care for lobar pneumonia. She had never smoked and had no previous lung disease or immunosuppression. At follow-up after discharge from the hospital, she was lethargic with a persistent cough but no weight loss or fever. CT of her thorax confirmed bilateral well-defined nodules up to 1 cm in diameter. Bronchoscopic washings grew mycobacteria, but the organism could not be speciated. Results of a percutaneous nodule biopsy were nondiagnostic. Surgical biopsy of the right lung found caseating granulomata, but culture was negative. At 7 months follow-up, a CT scan of her thorax showed no change in the nodules, but mild bronchiectasis had developed. Bronchoscopic lavage grew *M. lentiflavum* for the first time. In February 2009, she began ethambutol (800 mg), rifampin (450 mg 1×/d), and clarithromycin (500 mg 2×/d). Her symptoms improved, and she completed 18 months of treatment. Bronchoscopic washings posttreatment were ZN stain and AFB culture negative.

#### Case 3: Bronchiectasis

A 49-year-old Taiwanese woman who had never smoked sought care in 1998 for hemoptysis. She had moved to Australia 5 years earlier. Thoracic CT showed a right middle lobe infiltrate. Three sputum samples were culture negative for AFB. Transbronchial lung biopsy samples showed peribronchial granulomata but were culture negative. She received empirical quadruple therapy for tuberculosis. The cough continued but without hemoptysis. In 2004, a chest radiograph showed middle lobe and lingular bronchiectasis. Three sputum samples were AFB culture negative. Bronchoscopic washings were ZN negative but grew *M. lentiflavum*, thought to represent colonization. In 2007, an unspeciated NTM grew on 1 of 3 sputum specimens. By January 2009, the patient was well, with no exacerbations in the previous year and stable radiographic appearance.

#### Case 4: Cervical Lymphadenitis

A 20-month-old girl was examined for a 4-week history of bilateral cervical lymphadenopathy. The largest node (20 × 24 mm) was excised. Necrotizing granulomata were seen. *M. lentiflavum* was cultured. No antimycobacterial therapy was administered; she recovered fully.

### Nonsignificant Isolates

A 29-year-old woman underwent bilateral lung transplantation. Routine posttransplant bronchial washings grew *M. lentiflavum*. Despite immunosuppressive therapy, no further AFB were cultured from multiple samples in the subsequent 2.5 years.

In 7 patients (mean age 62 years, 4 male), 1 or 2 isolates of *M. lentiflavum* grew from sputum in the context of MAC disease or colonization. Four of these patients were concurrently treated for MAC; 1 had recently completed treatment; 2 received no treatment for NTM and continue under surveillance. All 7 had underlying lung disease (2 cavitatory, 5 bronchiectatic). In no instance was *M. lentiflavum* specifically treated. In addition, sputum of 3 patients grew *M. interjectum*, *M. fortuitum*, or *M .abscessus*.

From 3 otherwise healthy patients (40-year-old man, psoas abscess; 2-year-old girl, cervical lymphadenitis; 54-year-old man, neck abscess), *M. lentiflavum* and *Staphylococcus aureus* were cultured. All patients recovered fully after treatment with flucloxacillin with or without drainage. No samples were taken for histologic examination, but cytologic examination of a lymph node aspirate from the child showed lymphocytes, macrophages, neutrophils, and fragments of epithelioid histiocytes but no well-formed granulomas. From 2 other patients (35-year-old woman, chronic leg ulcer; 59-year-old woman, post thyroidectomy wound abscess), *M. lentiflavum* without *S. aureus* were cultured; the patients were treated with wound debridement and flucloxacillin. Biopsy samples showed no granulomata.

Most other isolates were cultured from respiratory samples. One isolate each was grown from ascitic fluid and blood. Three patients with cystic fibrosis (2 with mild disease, 1 lung transplant recipient) had 1 or 2 isolates each but no evidence of disease.

### Strain Types

**Figure 3:**
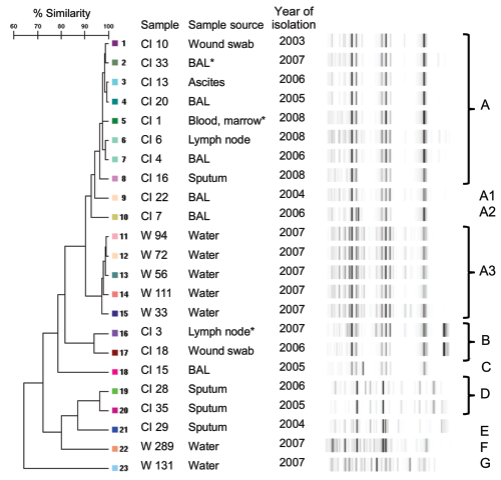
Dendrogram and virtual gel images representing rep-PCR fingerprint patterns of 16 human and 7 water isolates of *Mycobacterium lentiflavum*, Brisbane, Queensland, Australia, 2001–2008. CI, clinical isolate; W, potable water isolate; BAL, bronchoalveolar lavage. *Clinically significant isolate.

DiversiLab patterns were grouped into 7 rep-PCR profiles, A–G (Figure 3). The 8 clinical isolates of profile A showed 97%–99% similarity. This profile included 2 clinically significant isolates (cases 1 and 3) and 6 nonsignificant isolates (3 respiratory samples, 2 soft tissue samples, and 1 ascites sample). Two further pulmonary isolates (profiles A1 and A2) were ≈90% similar to the profile A isolates. The isolate from case-patient 2 was contaminated and could not be analyzed. The isolate from case-patient 4 (profile B) had 94% similarity to a nonsignificant isolate from soft tissue. These 2 isolates were from patients who lived 1,800 km apart.

Profile D comprised a pair of nonsignificant pulmonary isolates of 97% similarity. These isolates came from patients who lived within 80 km of each other, 450 km north of Brisbane. Profiles C and E were nonsignificant isolates and distinct from other rep-PCR profiles.

Five water sample isolates (profile A3) had 97%–99% similarity and shared 90% similarity with the clinical isolates of profiles A, A1, and A2. The other 2 water isolates (profiles F and G) were distinct from all other clinical and water isolates.

### Global Case Reports

In 30 cases of clinically significant disease published in English (Table A1), disease spectrum varied from cervical lymphadenitis (8 of 9 cases in children) to acute or chronic disease usually affecting lungs and pleura (infiltrates, cavities, nodules, effusions) but also arthritis/discitis, bone lesions, skin ulcers, and hepatosplenomegaly. The rapid onset of cervical lymphadenitis has been noted in many reports, usually with an excellent outcome from excision alone. The mean age of adults with nonlymphadenitis disease (20 cases) was 56 years (range 23–87 years), and they were evenly split between the sexes. Eleven case-patients had associated immunocompromise. Eleven were reportedly stable or improved at follow-up, 6 died, and 3 had uncertain outcomes.

## Discussion

*M. lentiflavum* disease can be difficult to diagnose, as the cases in this report exemplify. The clinical information we gathered was largely retrospective, which poses certain limitations; however, the case-patients 1 and 2 were current patients undergoing active treatment at the time of writing (June 2009). *M. lentiflavum* was isolated occasionally from patients colonized or undergoing treatment for MAC and in patients with *S. aureus* soft tissue infections. Certainly in some patients multiple NTM can grow at the same or different times, and *M. lentiflavum* may be no different in this respect. *M. lentiflavum* has been cultured from sputum containing MAC and from sputum containing *M. tuberculosis*, but these cases may represent colonization/contamination rather than infection (*23**,**28*). Concurrent isolation of *M. lentiflavum* and *S. aureus*, which probably represents contamination or colonization, has not been reported as far as we are aware. Co-infection of *S. aureus* and *M. tuberculosis* has been reported, possibly as superimposed staphylococcal infection in tuberculous tissue (*29**,**30*). Although no samples were taken for histology, cytologic examination of lymph node aspirate from the 2-year-old child with lymphadenitis is intriguing because the inflammatory cells were predominantly lymphocytes/macrophages with epithelioid histiocytes. Treatment using flucloxacillin with or without drainage affected a complete cure in all cases.

*M. lentiflavum* is a rare isolate and an unusual cause of disease in humans. As with other NTM, it can be isolated from contaminated samples: clinical significance should be assessed before any treatment is considered (*2*). In 2005, of 488 patients with pulmonary NTM isolates in Queensland, only 26.6% were considered to have clinically significant disease (*31*). The proportion was higher for *M. intracellulare* (39.4%), *M. avium* (33.3%), and *M. kansasii* (52.6%) and much lower for species traditionally thought to be more likely contaminants, e.g., *M. gordonae* (11.1%). In our study, isolates for 4 (11%) of 36 were clinically significant, similar to published estimates of 10%–21% (*16**,**22*). This proportion may be an underestimate given that we could not determine clinical significance in 4 patients. Worldwide, *M. lentiflavum* may be underreported and incorrectly identified as other, more familiar species, especially if access to molecular identification is limited.

*M. lentiflavum* has been isolated from water distribution samples. Torvinen et al. isolated NTM from up to 80% of sites across Finland (*32*); *M. lentiflavum* was the second most common species (38% of sites). Laboratory isolation of *M. lentiflavum* from clinical specimens in Finland has increased independently of speciation methods, but details of patients with disease are lacking (*33*). In South Korea, Lee et al. found mycobacteria in 26% of 84 drinking water sites. Sixty-five percent of isolates were *M. lentiflavum* (*34*). In our study, mycobacteria were isolated from 70% of sites, but *M. lentiflavum* from only 6.3%. The difficulties in isolating mycobacteria from potable water are well recognized and relate to mycobacterial growth characteristics and the need for specimen decontamination to reduce bacterial and fungal overgrowth. Decontamination reduces mycobacterial yields; hence, the prevalence of mycobacteria in potable water samples is believed to substantially underestimate the true figure (*8*). Culture-based techniques may be less sensitive than direct PCR. However, detecting mycobacterial DNA does not necessarily prove the presence of viable organisms that are able to cause infection; detection of *M. lentiflavum* by culture-based methods is noteworthy with respect to human health. Case-patient 1 had long-term intravenous access, which may have allowed direct exposure to contaminated water through illicit drug administration. In this report, we have geographically associated culture-positive water samples and clinical disease.

DiversiLab strain typing showed that profiles A and A3 were most prevalent among clinical and water isolates and shared ≈90% similarity. The criteria for interpreting rep-PCR typing results have been established for some mycobacterial species. For example, Cangelosi et al. found high concordance between restriction fragment-length polymorphism and rep-PCR, reporting 93% similarity as the cutoff value for clustered *M. tuberculosis* isolates and 92% for *M. avium* (*6*). The analysis of *M. abscessus* by Zelazny et al., the largest study of rep-PCR in NTM, used rep-PCR to successfully cluster *M. abscessus* strains that were clonally related by PFGE analysis (*35*). Four of the water samples constituting profile A3 and 1 unrelated strain (profile G) came from sites that shared a groundwater source. These findings suggest a dominant environmental strain closely related (90%), but not identical, to strains found in human specimens and as a cause of human disease. The theory of dominant local environmental strains is supported by the finding of a different strain type from 2 patients living near each other but 450 km from Brisbane (profile D).

Profile A contained clinically significant and nonsignificant isolates. Profile B also contained a pair of highly similar isolates (94%) of which 1 was clinically significant. Although the residential addresses of these patients were 1,800 km apart, nothing is known about the duration of residence or travel or work habits of these case-patients. Thus, the infection may not have been acquired locally. Conclusions cannot be drawn about the pathogenicity of different strains; a larger study is required to address this question.

The finding of different, less common strain types (profiles E, F, G) confirms the validity of using automated rep-PCR (DiversiLab) as a tool for strain typing this species. Variation in *M. lentiflavum* strain type has been demonstrated. Buijtels et al. (*20*) reported 55 *M. lentiflavum* isolates from 149 specimens obtained from 38 patients at 1 hospital in Zambia. Illness of 2 patients definitely fulfilled ATS/IDSA criteria for significant disease. Because this species is a rare cause of disease, the authors performed molecular identification on a subset of 12 isolates to investigate the possibility of laboratory contamination. Six strain types were identified; the Zambian strains clearly differed from comparator Dutch strains. The finding of a dominant strain probably represented the local endemic strain, but laboratory or point-of-collection contamination could not be entirely excluded. Because isolates in our study came from multiple laboratories statewide at different times, contamination is unlikely to explain their presence in multiple clinical specimens.

The optimal treatment for *M. lentiflavum* disease is not established; a wide variety of regimens has been used in previous case series. Although evidence does not support the use of any specific regimen, we achieved symptomatic and radiologic improvement in case-patients 1 and 2 with rifampicin/ethambutol/clarithromycin at 12 months and isoniazid/ethambutol/clarithromycin at 18 months, respectively. More detailed reporting of treatment regimes and outcomes will help establish optimum therapy.

## Appendix

**Table A1:** Summary of published cases of clinically significant *Mycobacterium lentiflavum* isolates*

Type of infection and reference†	Patient age/sex	Country	Organ/tissue involved	Immunocompromised	Concurrent conditions	Treatment	Outcome
Soft tissue/skin							
(*1*)	85 y/F	Germany	5-mo history thoracic discitis T9, T10. Biopsy showed granulomata, C+ after 22 d	No	Diabetes mellitus, congestive heart failure	[INH, RMP, PZA] 3 mo, then [INH/RMP] 6 mo	Marked symptomatic improvement at 2 mo
(*11*)	52 y/F	Spain	Arthritis, skin lesions, synovial fluid was sterile; synovial biopsy found granulomata, ZN+, C+ after 4 wk.	Yes, GC, cyclophosphamide	Antisynthetase syndrome	[INH, RMP, EMB, PZA] then [fusidic acid, LFX, CLR] for 1 wk before death	Condition worsened with weight loss, synovitis. Died after 4 mo
(*12*)	48 y/M	Spain	2 skin ulcers, 2-y history. Biopsy ZN+, C+	Yes	HIV for 15 yr, CD4+ 46, IVDU	[Highly active antiretroviral therapy, INH, EMB, LFX]	Lost to follow-up
Cervical lymphadenitis							
(*13*)	42 mo/M	Germany	10-d history, ZN+, C+, granulomata present	No	No	Excision	Well at 18 mo
(*13*)	33 mo/M	Germany	14-d history. ZN–, C+, granulomata present	No	No	Excision	Well at 18 mo
(*14*)	4 y/F	Italy	15-d history, biopsy scanty ZN+, C+	No	No	[INH, RMP] duration NS; excision	Well at 2 y
(*15*)	19 mo/M	Spain	10-d history, ZN+, C+, granulomata present	No	Asthma	[INH, RMP, PZA] duration NS; then excision	Recovered fully
(*16*)	4 y/M	Italy	No details	No	No	Excision	Well at 1 y
(*17*)	45 y/F	Italy	4-wk history. ZN–, C+ at 10 d, granulomata present	No	Severe periodontal disease	Excision	Well at 18 mo
(*18*)	18 mo/M	Italy	4-wk history. ZN+, C+ after 3 wk; recurrent lymphadenopathy at 3 mo in same position showed granulomata	No	No	Excision	Recurrent lymphadenopathy after 3 mo treated with excision
Queensland§	20 mo/F	Australia	4-wk history, biopsy ZN–, granulomata present, C+	No	No	Excision	Full recovery
Pulmonary							
(*14*)	58 y/M	Italy	Left upper lobe pulmonary infiltrates, pleural effusion, low-grade fever, weight loss. Pleural fluid and sputum C–; pleural biopsy showed granulomata, ZN–, C+ after 3 wk	Yes(rheumatoid arthritis)	Rheumatoid arthritis	[INH, RFB, EMB, PZA]	Stable at 4 mo. No improvement in computed tomography thorax appearance
(*16*)	61 y/F	Italy	Cough, fever, weight loss, right upper lobe nodular pulmonary infiltrates and adenopathy. Sputum ZN+, C+	No	Bronchiectasis since age 30 y	[RMP, INH, PZA] for “several months,” then [RFB, EMB, CLR, CIP]	5-y follow-up, sputum intermittently ZN+, C+; no improvement in radiology
(*19*)	71 y/M	Japan	Hemoptysis, bilateral pulmonary infiltrates, cavities right upper lobe. Sputum ZN+, C+ after 35 d(multiple specimens)	No	2-y treatment for pulmonary TB aged 30 y; smoker	[RMP, EMB, INH, PZA] 1 y, CLR also added, duration NS	3-y follow-up, sputum remained ZN+, C+, symptoms continued, CXR slowly progressed
(*20*)(case 6)	35 y/F	Zambia	4-wk history of cough, pleural effusion fluid C+.	No	No	[RMP, INH, PZA, EMB] duration NS	Improved. Duration of follow-up NS
(*21*)	67 y/F	Italy	Hemoptysis, low-grade fever, weight loss. Sputum ZN+, C+	No	Previous pulmonary TB, with fibrosis right upper lobe, chronic obstructive pulmonary disease	[INH, PZA, EMB, RMP] 3 mo: no effect: ceased. 2 y later, started CLR monotherapy, improved by 3 mo, but sputum still ZN+ [EMB, RFB, CIP] added for 2 wk	3-y follow-up. Poor compliance with drugs, intermittent hemoptysis, weakness, dyspnoea, sputum remained ZN+, CXR unchanged
(*22*)	49 y/M	United States	Fever, right upper lobe pulmonary noncavitary nodules, 2×/ sputum C+ at 27 d, bronchoalveolar lavage C–	Yes	Myelofibrosis on pegylated interferon-α2	[CIP 500 mg 2×/day, azithromycin 500 od, EMB 1 g od] unknown duration	Stable disease, lost to follow up; duration of follow-up NS
(*22*)¶	66 y/M	United States	Fever, neutropenia, necrotizing pneumonia, single sputum C+ at 28 d	Yes	Hematopoetic stem cell transplant for chronic lymphocytic leukemia, graft vs. host disease	[CLR 500 mg 2×/day, EMB 1 g od]	Died after 12 wk, septic shock from *Pseudomonas aeruginosa*. Uncertain significance of *M. lentiflavum*
(*23*)¶	28 y/M	Brazil	Cough, fever, reticulonodular infiltrate, PCP+, 1× sputum C–. 3 wk later high fever returned – 1× blood C+	Yes	Newly diagnosed HIV, PCP	[Streptomycin] duration NS(? 1 mo) GC, sulphamethoxazole–trimethoprim, zidovudine, didanosine	Died a few mos later, no further clinical details
(*23*)¶	64 y/F	Brazil	Pulmonary cavities, treated for ZN+ pulmonary tuberculosis for 6 y	Yes	Chronic pulmonary tuberculosis	Various including CLR, EMB, clofazimine, RMP and doxycycline. No details given.	Sputum persistently C+: 12/15 sputum samples grew *M. avium* complex, 2 grew *M. lentiflavum*. Died of uterine cancer
(*18*)	14 y/M	Italy	Fever, chest pain, pleural effusion, pneumothorax, multiple right upper lobe and right middle lobe nodules. Pleural fluid ZN+, C+ at 4 wk	Yes	Acute lymphoblastic leukemia, chemotherapy	[CLR, amikacin, ceftriaxone] 5 mo	Improved, CXR almost returned to normal
(*18*)	87 y/M	Italy	Bilateral pleural thickening, left pleural effusion, pulmonary opacities, pleural fluid ZN–, C+ at 3 wk	No	No	[LFX] monotherapy for 3 wk, then [CLR, LFX, RFB]	After 1 mo, pulmonary opacities resolved, effusion remained
(*24*)(case 2)	23 y/M	Greece	Pleural effusion, sputum C+	NS	NS	[INH, RMP, PZA, EMB] duration NS(? 6 mo)	Recovered
Queensland§	85 y/F	Australia	Cough, pulmonary nodules, mild bronchiectasis, bronchial washings ZN–, C+	No	No	[EMB 800 mg, RMP 450 mg, CLR 500 mg 2×/day]	Stable at 7 mo
Queensland§	49 y/F	Australia	Hemoptysis, bronchiectasis, bronchial washings ZN–, C+	No	No	No specific treatment given	Currently well, stable CXR at 11 y
Disseminated							
(*25*)	49 y/M	France	Fever, pulmonary infiltrates, 3× blood culture and bronchoalveolar lavage C+ after 4 wk	Yes	HIV for 7 y	[CLR, RFB, EMB] and antiretroviral drugs, at least 4-mo treatment, probably 9 mo	Fully recovered, died 3 y later of heart failure
(*16*)¶	45 y/M	Italy	Basal pulmonary infiltrate, T4 vertebra involvement, hepatic lesion. Empiric treatment given with clinical improvement. Liver biopsy C+, no other samples reported	Yes	HIV, non-Hodgkin lymphoma	[Antiretrovirals, RFB, CLR], then [RFB, CIP, EMB, CLR] then [RFB, CLR]	Well at 1 y. Hepatic lesion aspirated C+ but continued to grow; was later excised and proven to be non-Hodgkin lymphoma
(*20*)(case 1)	67 y/F	Zambia	8-wk duration hepatosplenomegaly, axillary lymphadenopathy; lymph node biopsy ZN+, C+; 2× Sputum C+	No	No	No treatment given	NS
Queensland§	43 y/F	Australia	4x blood cultures C+ after 15 d, 1x bone marrow ZN–, granulomata present, C+; hepatosplenomegaly, pulmonary nodules	Yes	HIV, hepatitis C, IVDU, systemic lupus erythematosus	[INH 300 mg, EMB 400 mg 2×/day, CLR 500 mg 2×/day] with reducing course of prednisone	Compliant with treatment, improving at 12 mo

*C+, culture positive; INH, isoniazid; RMP, rifampicin; PZA, pyrazinamide; ZN+, Ziehl-Neelsen smear stain–positive; GC, glucocorticoids; EMB, ethambutol; LFX, levofloxacin; CLR clarithromycin; IVDU, intravenous drug user; ZN–, Ziehl-Neelsen smear stain–negative; NS information not stated or unknown; RFB, rifabutin; right upper lobe; CIP, ciprofloxacin; TB, tuberculosis; CXR, chest radiograph; C–, culture negative; PCP, Pneumocystis pneumonia. †Excluded are 2 cases from articles written in Spanish(26,27). ‡Brackets indicate antimicrobial drugs used together as a single regimen. §Cases reported in this article. ¶Not definite cases of disease.
