# Errata—Vol. 17, No. 3

**DOI:** 10.3201/eid1709.C21709

**Published:** 2011-09

References were misnumbered in the Appendix Table of the article *Mycobacterium lentiflavum* in Drinking Water Supplies, Australia (H.M. Marshall et al.). The article has been corrected online (www.cdc.gov/eid/content/17/3/395.htm).

